# The Effects of Meditation, Yoga, and Mindfulness on Depression, Anxiety, and Stress in Tertiary Education Students: A Meta-Analysis

**DOI:** 10.3389/fpsyt.2019.00193

**Published:** 2019-04-24

**Authors:** Josefien J. F. Breedvelt, Yagmur Amanvermez, Mathias Harrer, Eirini Karyotaki, Simon Gilbody, Claudi L. H. Bockting, Pim Cuijpers, David D. Ebert

**Affiliations:** ^1^Department of Psychiatry, Academic Medical Center, Amsterdam University Medical Center, Amsterdam, Netherlands; ^2^Research Department, Mental Health Foundation, London, United Kingdom; ^3^Department of Clinical, Neuro-, and Developmental Psychology, Amsterdam Public Health Research Institute, Vrije Universiteit Amsterdam, Amsterdam, Netherlands; ^4^Department of Psychology, Clinical Psychology and Psychotherapy, Friedrich-Alexander-University Erlangen-Nuremberg, Erlangen, Germany; ^5^Mental Health and Addictions Research Group, Department of Health Sciences, University of York, York, United Kingdom

**Keywords:** tertiary education, meditation, yoga, mindfulness, anxiety, depression, stress, university

## Abstract

**Background:** Meditation, yoga, and mindfulness are popular interventions at universities and tertiary education institutes to improve mental health. However, the effects on depression, anxiety, and stress are unclear. This study assessed the effectiveness of meditation, yoga, and mindfulness on symptoms of depression, anxiety, and stress in tertiary education students.

**Methods:** We searched Cochrane Central Register of Controlled Trials (CENTRAL), PubMed, PsycINFO and identified 11,936 articles. After retrieving 181 papers for full-text screening, 24 randomized controlled trials were included in the qualitative analysis. We conducted a random-effects meta-analysis amongst 23 studies with 1,373 participants.

**Results:** At post-test, after exclusion of outliers, effect sizes for depression, g = 0.42 (95% CI: 0.16–0.69), anxiety g = 0.46 (95% CI: 0.34–0.59), stress g = 0.42 (95% CI: 0.27–0.57) were moderate. Heterogeneity was low (*I*^2^ = 6%). When compared to active control, the effect decreased to g = 0.13 (95% CI: −0.18–0.43). No RCT reported on safety, only two studies reported on academic achievement, most studies had a high risk of bias.

**Conclusions:** Most studies were of poor quality and results should be interpreted with caution. Overall moderate effects were found which decreased substantially when interventions were compared to active control. It is unclear whether meditation, yoga or mindfulness affect academic achievement or affect have any negative side effects.

## Introduction

### Rationale

Every 12 months, between 7 and 16% of students in tertiary education experience a mood or anxiety disorder and a further 30% of students report experiencing moderate to severe levels of stress (1–4).

It is important to tackle poor mental health early as unattended symptoms can contribute to poorer clinical outcomes such as an increased risk of developing a clinical diagnosis or relapse (5). When in distress, few students seek or receive treatment (6). This is due to several barriers such as stigma and lack of awareness of services (6).

Mindfulness, meditation, and yoga have been coined as a non-stigmatizing alternative to traditional mental health support. They are highly popular tools at tertiary education institutes and used for stress reduction, improve productivity and general mental health (7). Yoga, mindfulness, and meditation are part of a suite of interventions called mind-body interventions (8). They are closely related practices and share underlying common principles and therapeutic elements grounded in religion and spirituality (9–12).

The most commonly known and offered mindfulness program is Mindfulness-Based Stress Reduction (13). MBSR includes a set of specific mindfulness practices including focused attention on the breath, “body-scanning,” prosocial meditation (e.g., loving kindness and compassion), and gentle hatha yoga. MBSR is different from Mindfulness-Based Cognitive Therapy (MBCT) as it includes cognitive therapeutic elements such as cognitive restructuring and is aimed at reducing depressive relapse (14). Yoga is defined as a variety of practices which includes postures, breathing exercises, meditation, mantras, lifestyle changes spiritual beliefs, and/or rituals (15). A frequently practiced form of yoga is Hatha Yoga, which includes asanas (postures, pranayama (breathing exercises) and meditation, usually integrated throughout the practice (16).

Several reviews have been conducted to assess the effects of mindfulness and yoga-based interventions on a range of outcomes and populations. Reviews assessing the evidence for yoga have covered PTSD (17), depression (18, 19), anxiety (20), and physiological measures of stress (21, 22). For mindfulness and meditation interventions, reviews have assessed mood, and general functioning of students (23), employee mental health (24), stress management (25, 26), depression, stress and wellbeing (27), recurrent depression (28), and anxiety (27, 29). The reviews are wide-ranging in their conclusions and offer mixed results. Whilst the majority of reviews suggest preliminary evidence for their effectiveness, the authors often comment on the need for more rigorous research in this area.

The debate about the effects of these alternative medicine interventions thus remains. A recent review by Goyal et al. (27) found a pool of low-quality studies, with limited evidence for effect especially when compared to specific active treatment control conditions such as behavioral therapies, relaxation interventions, or exercise.

It is important to address the effects of these interventions for students, clinicians and commissioners to make evidence-based decisions about the provision of mental health support at university. Whilst widely accessed, it is unclear whether yoga, mindfulness, or meditation have a beneficial effect on mental health or academic achievement in young adults beyond placebo.

### Objectives

This systematic review and meta-analysis aims to study the effectiveness of both yoga and mindfulness-based interventions on stress, depression, anxiety, and academic achievement for students in tertiary education.

### Research Question

What are the effects of mindfulness, meditation, and yoga on depression, anxiety stress and academic achievement in tertiary education students vs. control?

## Methods

### Study Design

This study utilized a systematic review and meta-analysis in order to answer the above research question.

### Participants, Interventions, Comparators, Outcomes

Included studies were randomized controlled trials, published in English, in which a meditation, yoga or mindfulness intervention was compared to an active or non-active control group (wait-list, treatment-as-usual, placebo or active treatment control). Participants had to be enrolled in tertiary education when they were randomized into treatment group in the study (i.e., a university, college or other postsecondary higher education). Studies in which measured depression, anxiety, stress (i.e., Beck Depression Inventory (BDI), the State-Trait Anxiety Inventory (STAI), academic achievement (i.e., productivity, GPA, absenteeism) or a combination of these, as measured via a validated questionnaire were included. Additionally, enough information needed to be provided to calculate the effect size. We contacted authors when we were unable to calculate effect sizes based on the information provided in the paper.

### Systematic Review Protocol

The procedure for this systematic review is outlined according to the Preferred Reporting Items for Systematic Reviews and Meta-Analyses Protocol (PRISMA-P) guidelines (30).

### Search Strategy and Data Sources

Publications were identified by searching Cochrane Central Register of Controlled Trials (CENTRAL), MEDLINE, and PsycINFO by combining terms (text words, MeSH terms and subject headings) on (1) student population, (2) psychological interventions, (3) mental health or academic outcomes, and (4) randomized controlled trials. We conducted the searches on 27.04.17. We included studies with any date of publication which were either published, under review or “in press.” We contacted authors of study protocols that were suitable for inclusion to assess whether any unpublished results were available for inclusion. The search string can be found in Appendix 1 in Supplementary Material. Additionally, we searched for references in prior meta-analyses and included studies until 02.03.18.

### Study Selection and Data Extraction

#### Study Selection

**Figure 1:**
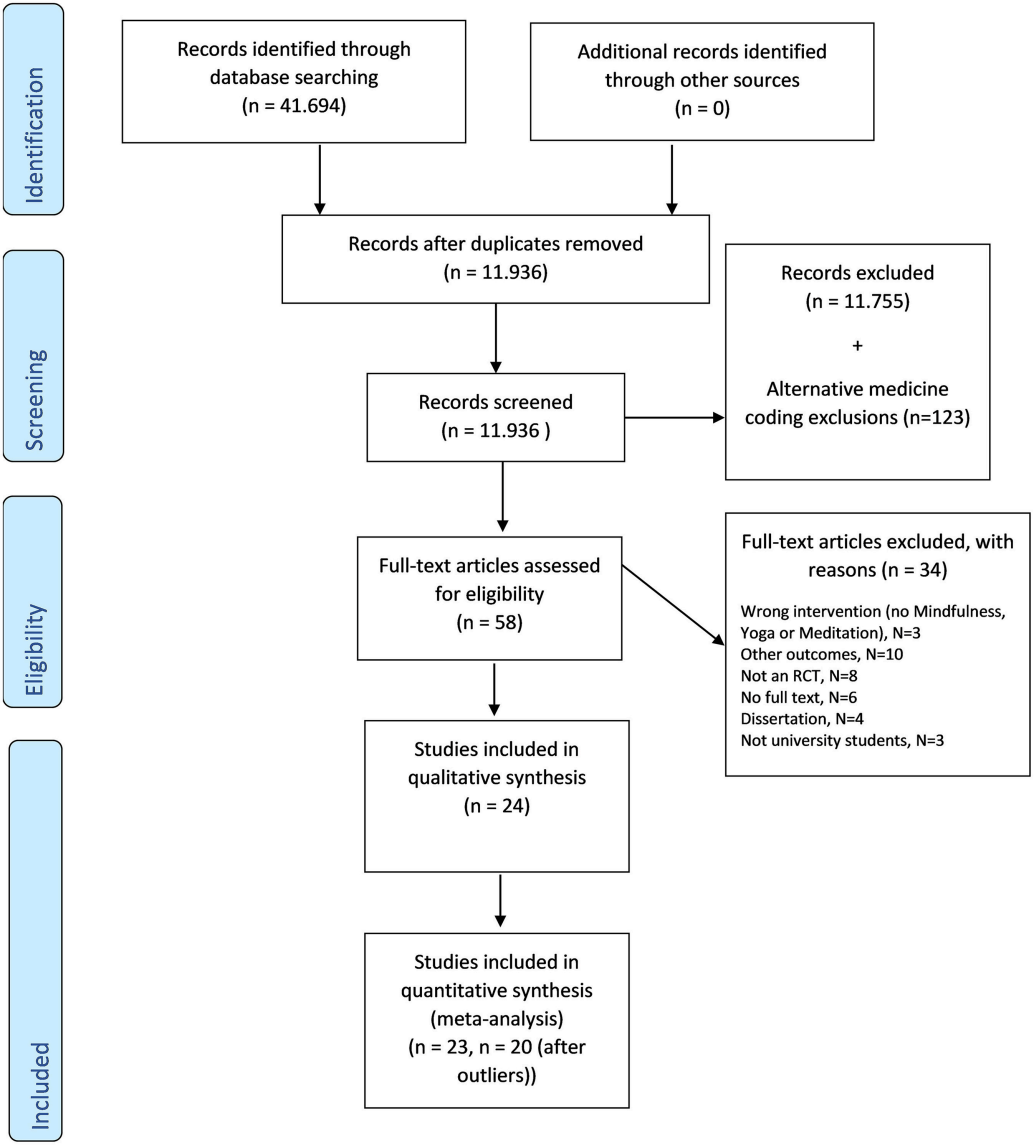
Prisma-P flow chart.

Titles and abstracts of articles identified through the database search were screened by two independent researchers. The researchers coded all retrieved results to separate categories, including one pool of “alternative medicine” studies. Please see Appendix 2 for an overview of study coding procedures. From the alternative medicine study pool, studies were coded as yoga, mindfulness or meditation and these were then retrieved for full-text screening. In this second step full texts of all studies that were deemed suitable were retrieved and reviewed for eligibility by two researchers (JB and YA). When there was any disagreement the authors convened for a discussion; senior researchers (DE or PC) were consulted if disagreement could not otherwise be resolved. Figure 1 shows the PRISMA-P flow chart.

### Data Extraction

A standardized electronic data collection form following Cochrane Good practice guidance was used to extract data. The following variables were extracted: (1) bibliographical data, (2) study design, (3), sample characteristics (e.g., gender, % female, ethnicity), (4), intervention type (mindfulness, MBSR, yoga, meditation) (5) exposure to the intervention [e.g., duration of each session, duration of home practice, length of intervention period (weeks)]. Exposure was calculated as the duration of meditation each week (in session and home practice) (in minutes) × length of intervention (weeks). (6) Setting (country), (7) intervention modality (e.g., face to face, online, group setting) (9) outcomes (depression, anxiety, stress, academic achievement), and (10) drop-out and handling of missing data. Two reviewers (JB and YA) independently extracted the study data and resolved any discrepancies by consulting a third reviewer (MH). When studies conducted assessments for the above outcomes during an exam and in non-exam settings, we extracted the non-exam setting only. Please see Table 1 for an overview of interventions and intervention descriptions.

**Table 1:** Definitions of intervention categories.

**Name**	**Description**
Mindfulness	According to Creswell (31), mindfulness is “a process of openly attending, with awareness, to one's present moment experience.” Creswell (31). A frequently practiced form of Mindfulness is Mindfulness Based Stress Reduction [MBSR] (13). MBSR includes a set of specific mindfulness practices including focused attention on the breath, “body-scanning,” prosocial meditation (e.g., loving kindness and compassion), and gentle hatha yoga.
Meditation	According to Shapiro et al. (32) meditation is defined as “a family of self-regulation practices that focus on training attention and awareness in order to bring mental processes under greater voluntary control and thereby foster general mental well-being and development and/or specific capacities such as calm, clarity, and concentration.” Walsh and Shapiro (33).
Yoga	According to Birdee et al. (15), yoga is defined as a variety of practices which includes postures, breathing exercises, meditation, mantras, lifestyle changes spiritual beliefs and/or rituals (15). A frequently practiced form of yoga is Hatha Yoga, which includes asanas (postures, pranayama (breathing exercises) and meditation, usually integrated throughout the practice Riley (16).

### Risk of Bias

Two researchers assessed the risk of bias (JB and YA), which was extracted using an approach based on the Cochrane Collaboration risk of bias assessment criteria for RCTs described by Furlan et al. (34). The criteria were: (1) random sequence generation, (2) allocation concealment, (3) blinding of participants, (4) blinding of personnel, (5) blinding of outcome assessors, (6) incomplete outcome data, (7) selective outcome reporting (which was established searching for the protocol on PubMed and Google Scholar and assess discrepancies between included outcomes and reported outcomes, if no protocol was available we examined the methods for any unreported outcomes), (8) serious flaw. Studies were scored as “low,” “high,” or “unclear” risk of bias on each of these domains. If the researchers scored six criteria as “low” and if there were no serious flaws detected, the study was scored to have a low risk of bias.

### Researcher Allegiance

For researcher allegiance assessment, the reprint approach with criteria operationalized to assess the “belief [of the investigators] in the superiority of a treatment [and] … the superior validity of the theory of change that is associated with the treatment” was applied (35). A six-point rating scale indicating various degrees of risk for researcher allegiance derived and adapted from Wampold et al. (36) was used.

### Convenience Sampling Rating

We defined studies as employing convenience samples when either: (1) the sample only contains students of the investigators institute, (2) the sample was recruited through an internal study recruitment system used for the recruitment of research participants, (3) course credit was given, or (4) the article states a convenience sample was used.

### Data Analysis

Comprehensive Meta-Analysis (CMA) software (Biostat, Inc.) and STATA version 15 were used for the analysis (37). For each study included in the quantitative analysis, between-group effect sizes between intervention and control group at post-intervention were calculated (Hedges' *g*). For the main outcome analysis, three separate analyses were conducted to quantify the effects of studies on depression, anxiety, and stress. In the case of multiple treatment groups, the mean effect sizes were pooled for each study. When studies only recorded outcomes taken during an exam, we extracted these and conducted a sensitivity analysis to assess whether results were comparable.

A random effects model was used to pool effect sizes as we expect considerable heterogeneity amongst studies (38). To improve clinical interpretation, *g*-values were converted into the numbers-needed-to-treat (NNT) using Furakawa's method (39). The assumed response rate (50% reduction in symptoms) in the control group was 19% (40). The response rate was estimated from studies that assessed psychotherapy for depression and we assumed similar rates could be achieved in these studies. The NNT reflects the number of participants that need to receive the intervention in order for a positive outcome for one participant (41). 95% confidence intervals and two-sided *P*-values for each outcome were calculated.

The *I*^2^ statistic was used to determine heterogeneity (42). *I*^2^ heterogeneity of 25% was deemed low, 50% moderate, and 75% as substantial heterogeneity (43). The 95% confidence intervals were calculated using the STATA module *heterogi* (44). In this, a non-central chi-square based approach was used. Sensitivity analyses were conducted to assess whether study quality was related to effect sizes by comparing studies indicating a low risk of bias with all other studies. In addition, we examined the association between researcher allegiance, the use of a convenience sample and the treatment effects. Publication bias was tested by inspection of the funnel plot on primary outcome measures. Egger's test, a test for asymmetry of the funnel plot, was performed to attain quantitative results on publication bias (45).

When funnel plot inspection or Egger's test suggested the presence of bias, we applied the Duval and Tweedie trim and fill-procedure. This procedure estimates the number of missing studies and adjusts the effect size accordingly to attain a more unbiased estimate of the pooled effect size (46).

### Subgroup Analysis and Meta-Regression

Subgroup analyses and bivariate regression analyses were conducted to explore the following moderators; type of control, researcher allegiance, risk of bias, country of study, intervention type, exposure of intervention [duration of meditation each week (in session and home practice) (in minutes) x length of intervention (weeks)], and delivery of intervention (therapist, group, self-help).

## Results

### Selection and Inclusion of Studies

After screening 11,936 abstracts, 181 studies were retrieved and coded. Of these 181 studies, 58 studies covered a meditation, yoga or mindfulness intervention. Subsequently, we identified 24 studies as fitting our inclusion criteria, for further detail on study selection, please see Figure 1.

### Study Characteristics

Out of 24 included studies in both the quantitative and qualitative analysis, nine were conducted on the North American continent, 12 in Asia, and three in Europe. Eighty-three percent of participants were female. All studies used a “convenience sample” and most studies were conducted with participants from a medical faculty (*N* = 14). With regards to symptom levels in the sample, only one study excluded participants with low scores on the Penn State Worry Questionnaire (47). All other studies were aimed at a healthy or subclinical population. A further overview of study characteristics can be found in Table 2.

**Table 2:** Study characteristics.

**Study**	**Country**	**Target group**	***N***	**Intervention**	**Control**	***N* intervention**	***N* Control**	**Exam setting**	**Measure**	**Follow-up**	***N* groups**
Malathi and Damodaran (48)	India	Medical university students	50	Yoga	Wait list	25	25	Y	STAI	No	2
Tloczynski (49)	USA	University students	10	Meditation	No treatment	3–4	3	N	CAS	2 weeks, 4 weeks	3
Chang (50)	USA	Music major and graduate students	19	Meditation	Wait list	9	10	Y	PAI	No	2
Nidich et al. (51)	USA	University students	207	Meditation	Wait list	93	114	N	POMS	No	2
Gopal et al. (52)	India	MBSS students	60	Yoga	No treatment	30	30	Y	GARS, STAI-S	No	2
Kim (53)	Korea	Nursing students	30	Yoga	No treatment	15	15	N	ISSCS	No	2
Nemati (54)	Iran	MA post graduate students	107	Yoga	No treatment	58	49	Y	TAS	No	2
Shankarapillai et al. (55)	India	Dental students	100	Yoga	Active control (Psycho-education)	50	50	Y	STAI	No	2
Sharma et al. (56)	India	Medical, nursing and allied medical sciences	90	Fast pranayama (Yoga) OR slow pranayama (Yoga)	No treatment	30–30	30	N	PSS	No	3
Erogul et al. (57)	USA	1st year medical students	57	Mindfulness Based Stress Reduction	No treatment	28	29	N	PSS	6-months	2
Song and Lindquist (58)	Korea	Nursing students	44	Mindfulness Based Stress Reduction	Wait list	21	23	N	DASS-21	6-months	2
Esch et al. (59)	Germany	University students	43	Mindfulness Based Stress Reduction	Wait list	24	19	N	PSS	No	2
Shapiro et al. (32)	USA	Premedical and medical students	78	Mindfulness Based Stress Reduction	Wait list	37	41	Y	STAI, SCL-90-D	No	2
van Dijk et al. (60)	Netherlands	First year clinical clerkship students	167	Mindfulness Based Stress Reduction	No treatment	83	84	N	BSI	3, 7, 12, 15, 20-months	2
Paholpak et al. (61)	Thailand	Fifth year medical students	58	Meditation	No treatment	30	28	N	SCL-90	No	2
Call et al. (47)	USA	Psychology students	91	Yoga or Mindfulness	Wait list	29–27	35	N	DASS-21	No	3
Danilewitz et al. (62)	Canada	Pre-clerkship students	30	Mindfulness	Wait list	15	15	N	DASS-21	No	2
Greeson et al. (63)	USA	Undergraduate, graduate, professional students	90	Meditation (and Mindfulness)	Wait list	45	45	N	PSS	No	2
Kvillemo et al. (64)	Sweden	University students	76	Mindfulness Based Stress Reduction	Active control (Expressive writing)	40	36	N	CES-D	No	2
Chen et al. (29)	China	Nursing students	60	Meditation (and Mindfulness)	No treatment	30	30	N	SAS, SDS	No	2
Kang et al. (65)	Korea	Nursing students	32	Mindfulness (and Meditation)	No treatment	16	16	N	STAI, BDI, PWI-SF	No	2
Ratanasiripong et al. (66)	Thailand	Nursing students	89	Meditation (and Mindfulness)	Active control (Biofeedback training)	29–29	31	N	STAI-S, PSS	No	2
Shearer et al, (67)	USA	Psychology students	74	Mindfulness Based Stress Reduction	No treatment or active control (Dog therapy)	27–25	22	N	STAI-S, BDI	1 and 2 weeks	3
Yazdani et al. (68)	Iran	Nursing students	38	Yoga	No treatment	19	19	N	GHQ	4-weeks	2

*BDI, Beck Depression Inventory; BSI, Brief Symptom Inventory; CAS, College Adjustment Scale; CES-D, The Center for Epidemiologic Studies Depression Scale; DASS-21, Depression Anxiety Stress Scale-21; GARS, Global Assessment Of Recent Stress Scale; GHQ, Goldberg and Hiller's General Health Questionnaire; LSSCS, Life Stress Scale for College Students; PAI, Performance Anxiety Inventory; POMS, Profile of Mood States; PSS, Perceived Stress Scale; PWI-SF, Psychosocial Well-being Index-Short Form; SAS, Self-Rating Anxiety Scale; SCL-90, Symptom Checklist-90; SCL-90-D, Symptom Checklist-90-Depression Scale; SDS: Self-Rating Depression Scale; STAI, State-Trait Anxiety Inventory (T, Trait; S, State); TAS, Test Anxiety Scale; VAS-A, Visual Analog Scale for Anxiety*.

Out of 24 studies, the average rating of research allegiance was 2.63 and three studies scored 5/5. Eight studies provided information on ethnicity, of these, most participants were Caucasian (68%, *N* = 484), followed by Asian (12%, *N* = 88) and African/ African American (10%, *N* = 72).

The average length of the intervention was ~7 weeks. On average, participants practiced meditation yoga or mindfulness for 153 min each week, totalling to overall average exposure at 19 h and 36 min. All studies but two were offered in a group setting, with two offered as self-help, one of these approaches was an internet-based intervention. Four treatment-control comparisons utilized an active control, 10 studies used wait-list control and 10 provided no treatment. Please see Table 3 for a further specification of intervention characteristics.

**Table 3:** Intervention characteristics.

**Study**	**Intervention type**	**Description of intervention**	**Guidance**	**Delivery**	**Average duration per week (min.)**	**Length (weeks)**	**Exposure**
Call et al. (47)	Hatha yoga (Yoga) Body scan (Mindfulness)	Psychoeducation, breathing exercises, awareness, and acceptance	Guided	Group	45	3	135
Chang (50)	Meditation	Psychoeducation, discussion about personal meditation experiences, discussions about personal meditation experiences.	Guided	Group	225	8	1,800
Chen et al. (69)	Meditation	Psychoeducation, breathing, awareness of thoughts, and feelings	Guided	Group	210	1	210
Danilewitz et al. (62)	Mindfulness	Common problems of medical students (work-life-balance, perfection etc.), being mindful in clinical experiences	Guided	Group	75	8	600
Erogul et al. (57)	Mindfulness based stress reduction	Psychoeducation on stress, body scan, breathing	Guided	Group	253.5	8	2,028
Esch et al. (59)	Mindfulness based stress reduction	Psychoeducation on stress and related topics, relaxation exercises, retrospection	Guided	Group	120	8	960
Gopal et al. (52)	Yoga	Yogic prayer, micro and macro exercises, asanas postures, pranayama, and dhyana meditation	Guided	Group	245	12	2,940
Greeson et al. (63)	Meditation	Koru meditation. Breathing exercises, walking meditation, guided imagery, eating meditation	Guided	Group	145	4	580
Kang et al. (65)	Mindfulness	Body scan, breathing meditation, walking meditation, gratitude exercises	Guided	Group	90	8	720
Kim et al. (53)	Yoga	Breathing and relaxation exercises, mediation	Guided	Group	420	12	5,040
Kvillemo et al. (64)	Mindfulness	Theoretical foundations of mindfulness regarding relaxation, meditation, and the body-mind connection. Each weekly module consisted of a few pages of text (i.e., the lecture) and a set of exercises	Unguided	Online self-help	226.25	8	2,130
Malathi, and Damodaran (48)	Yoga	Yogic prayer	na	na	180	12	2,160
Nemati (54)	Yoga	Pranayama Yoga. Sitting quietly, breathing techniques, positive mantras	Guided	Group	Unclear	One full semester	Unclear
Nidich et al. (51)	Meditation	Transcendental Meditation, psychoeducation about TM and discussion about effectiveness	Guided	Group	Unclear	12	Unclear (at least 460)
Paholpak et al. (61)	Meditation	Breathing Meditation. mindful awareness, breathing exercises	Guided	Group	145	4	580
Ratanasiripong et al. (66)	Meditation	Psychoeducation on Vipassana meditation	Guided	Group + self-help	Unclear	4	Unclear
Shankarapillai et al. (55)	Yoga	Yoga postures, breathing exercises, guided relaxation	Guided	Group	60	1	60
Shapiro et al. (32)	Mindfulness based stress reduction	Sitting Meditation, body scan, Hatha Yoga, loving kindness, and forgiveness mediation	Guided	Group	150	7	1050
Sharma et al. (56)	Yoga (2 types)	Fast pranayama: various rapid breathing techniques, relaxation techniques; slow pranayama: slow breathing techniques, relaxation techniques	Guided	Group	90	12	1,080
Shearer et al, (67)	Mindfulness	Breathing exercises, stretching and balancing exercises, psychoeducation on stress	na	Group	60	4	240
Song and Lindquist (58)	Mindfulness based stress reduction	Body scan, sitting meditation, Hatha yoga, mindful walking, standing, and eating	Guided	Group	120	8	960
Tloczynski (49)	Meditation	Opening up meditation, attending uncritically	Guided	na	167	4	668
van Dijk et al. (60)	Mindfulness based stress reduction	Interactive presentation each week related to the session theme (e.g., awareness of stress)Recognizing Automatic Behavior, Influence of Perception, Recognizing Boundaries, Awareness of Stress, Communication, work-life balance	Guided	Group	120	8	960
Yazdani et al. (68)	Yoga	Laughter yoga. Relaxation techniques, breathing exercises, laughter yoga techniques	Guided	Group	120	4	480

In two comparisons symptom scores were higher in the intervention group at post-test. In one case this was when the intervention was compared to an inactive control (69). In the other, the intervention performed worse compared to an active control (66). No studies reported any further adverse effects.

### Synthesized Findings

In the quantitative analysis, we included a total of 23 studies. The studies included 1,373 participants with 660 in the intervention and 713 in control. For the quantitative analysis, we could not include academic outcomes as there were only two eligible studies reporting these (54, 61). The study by Paholpak et al. (61) assessed breathing meditation on memory, academic functioning and psychiatric symptoms in medical students (*N* = 58). No significant difference was found between intervention and control group (effect size and significance here). Nemati (54) did find a significant difference between intervention (pranayama yoga) and control (*N* = 107) on academic functioning (effect size and significance here). Test anxiety (i.e., anxiety related to performing a particular test) was measured in two studies (50, 54). We thought the sample too small to pool in a sensitivity analysis and a further analysis was not conducted.

The overall post-treatment effect in the 23 comparisons between yoga and mindfulness-based therapies and control groups was g = 0.61 (95% CI: 0.40–0.81) with an NNT of 5 (please see Table 4). The heterogeneity was high (*I*^2^ = 74%, 95% CI: 61–83). Three studies were potential positive outliers with an extremely high effect size (g >1) (53, 55, 56). After exclusion of these studies, the effect size decreased to g = 0.42 (95% CI: 0.31–0.52), NNT = 7 for depression, anxiety, and stress combined. Heterogeneity also decreased substantially (*I*^2^ = 6%, 95% CI: 0–40).

**Table 4:** Effects of meditation based interventions on depression, anxiety, and stress compared to control with Hedges *g*.

		***N comparisons***	***g***	**95% CI**		***I^**2**^***	**95% CI**	***P*^a^**	**NNT**
All studies		23	0.61	0.40	0.81	74	61–83	–	5
One effect per study (highest)		20	0.45	0.34	0.56	0	0–48	0.00^*^	7
One effect per study (lowest)		20	0.41	0.29	0.54	16	0–51	0.00^*^	8
Extreme positive outliers excluded Kim (53, 55, 56)		20	0.42	0.31	0.53	5	0–40	0.00^*^	7
Follow-up (1 and 24 months)		6	0.39	0.17	0.61	11	0–77	0.00^*^	8
**SUBGROUP ANALYSIS**									
Symptom outcomes	Depression	10	0.42	0.16	0.69	62	24–81	0.00^*^	7
	Anxiety	15	0.46	0.34	0.59	0	0–54	0.00^*^	7
	Stress	10	0.42	0.27	0.57	5	0–64	0.00^*^	7
Theory	Yoga	4	0.68	0.41	0.96	0	0–85		4
	Mindfulness	4	0.40	0.10	0.69	0	0–85		8
	Meditation	8	0.37	0.19	0.55	13	0–68		9
	MBSR^b^	8	0.36	0.18	0.54	2	0–55	0.23	9
Risk of bias	High	18	0.43	0.30	0.55	0	0–50		7
	Low	2	0.39	0.09	0.68	73	n.a.	0.80	8
Exam setting	Exam	4	0.73	0.45	1.01	0	0–85		4
	Non-exam	16	0.36	0.25	0.48	0	0–52	0.02^*^	9
Continent	America	9	0.49	0.34	0.64	31	0–65		6
	Asia	8	0.45	0.27	0.64	0	0–69		7
	Europe	3	0.13	−0.13	0.39	0	0–90	0.06	27
Control	Active	4	0.13	−0.18	0.43	0	0 −85		27
	No treatment	10	0.39	0.22	0.55	0	0–62		8
	Wait-list control	10	0.52	0.36	0.67	0	0–62	0.08	6

a p < 0.05,

b MBSR, Mindfulness Based Stress Reduction.

Five studies had more than two groups. Three studies used a second “active control” (relaxation, bio-feedback or dog therapy) condition aside from a no treatment control (49, 66, 67) and one study included two versions of yoga (56). A further study compared the effects of mindfulness or yoga techniques to control (47) (hatha yoga and body scan vs. wait-list control). Including multiple groups in the analysis may artificially reduce heterogeneity and thus introduce bias. To address this, we conducted an analysis where we first only included the study with the strongest effect size. A second analysis only included the lowest effect size. As Table 4 shows, effect sizes were similar, and heterogeneity increased (*I*^2^_high_ = 0% (95% CI: 0–48%), *I*^2^_low_ = 16% (95% CI: 0–51).

### Effects of Mindfulness and Yoga Interventions on Depression, Anxiety and Stress

**Figure 2:**
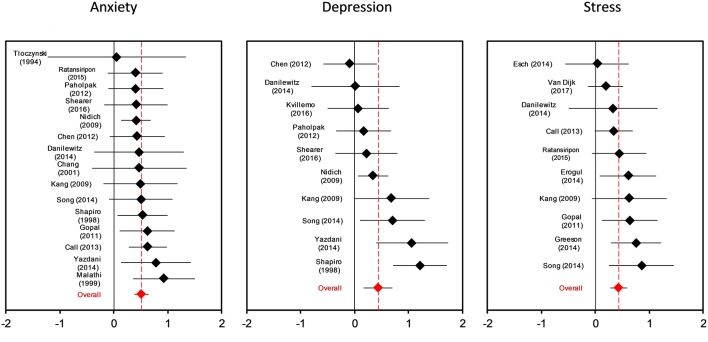
Forest plot for intervention effects on anxiety, depressing and stress symptoms with Hedges g.

Ten studies reported a depression outcome, 15 anxiety, and 10 stress. Figure 2 shows three forest plots on the separate outcome categories. Considering outcomes for depression, anxiety, and stress separately, the mean effect size for depression was g = 0.42 (95% CI: 0.16–0.69, *I*^2^ = 62%, 95% CI: 24–81), anxiety g = 0.46 (95% CI: 0.34–0.59, *I*^2^ = 0%, 95% CI: 0–54), and stress g = 0.42 (95%CI: 0.27–0.57, *I*^2^ = 5%, 95% CI: 0–64), respectively. There was no significant difference between these outcome categories in subgroup analysis (*p* = 0.41). Because depression, anxiety, and stress are highly interrelated and moderator characteristics were evenly distributed across outcomes, we conducted further subgroup analyses with pooled effect sizes across depression, anxiety, and stress outcomes.

### Long Term Follow-Up

Six studies provided long term follow-up data (i.e., any assessment after post-intervention) ranging between 1 and 24 months. The pooled effect size was small to medium g = 0.39 (95% CI: 0.17–0.61), and heterogeneity was low (*I*^2^ = 11%, 95% CI: 0–77).

### Subgroup Analysis

We conducted several subgroup analyses, for all results please see Table 4. Stronger effects were found when questionnaires were taken during an exam setting. When compared to active and inactive controls, the continent in which the study was conducted was not associated with effect size (*p* = 0.06). However, when we compared the effects to no-treatment control, we did identify a significant effect (*p* = 0.03), with studies conducted in Asia *g* = 0.54 (95% CI: 0.34–0.74) yielding strongest effects compared to America *g* = 0.49 (95% CI: 0.34–0.64), and Europe *g* = 0.13 (95% CI: −0.13 to −0.39). A subgroup analysis which compared yoga, mindfulness meditation, and MBSR did not find any significant subgroup differences between the intervention types.

### Meta-Regression Analyses

We conducted regression analyses on research allegiance, exposure to intervention, and overall Risk of Bias score. As a result, we did not find any significant explanatory value in these variables. Research allegiance (coefficient: 0; 95% CI: −0.11 to 0.08; *p* = 0.76) or the exposure to mindfulness, meditation or yoga (coefficient: 0; 95% CI: 0–0; *p* = 0.50). A further bivariate regression analysis on total RoB score and effect size did not identify significant subgroup differences either (coefficient: 0; 95% CI: −0.08 to 0.09; *p* = 0.91).

### Risk of Bias

Overall, only one study was scored to have a low risk of bias (60). Most studies had an unclear risk of selection bias. Six studies reported adequate sequence generation, the remaining studies did not report methods or used inappropriate methods. Adequate blinding of participants and personnel was rare. None of the studies adequately blinded participants, which is common amongst psychological research trials. Two studies reported that outcome assessors were blinded and three reported blinding of personnel. Only four studies reported conducting an ITT analysis. In other studies, this was either unclear or a completer analysis was conducted. See Table 5 for an overview of the risk of bias assessments.

**Table 5:** Risk of Bias.

**Study**	**Random sequence generation (Selection bias)**	**Allocation concealment (Selection bias)**	**Blinding (Participants) (Performance bias)**	**Blinding (Personnel) (Performance bias)**	**Blinding (Outcome Assessors) (Detection bias)**	**Incomplete Data (%) (Attrition bias)**	**Incomplete data (ITT Analyses) (Attrition bias)**	**Selective outcome reporting (Reporting bias)**	**Similar groups (Other bias)**	**Compliance (Other bias)**	**Identical post timing (Other bias)**	**Cointerventions (Other bias)**
Call et al. (47)	Unclear	Unclear	High	High	High	Unclear	High	Unclear	High	Unclear	Low	Low
Danilewitz et al. (62)	Unclear	Unclear	High	Unclear	Unclear	High	Unclear	High	Unclear	High	Low	Unclear
Esch et al. (59)	Unclear	Unclear	High	Low	High	Low	Unclear	Unclear	Low	Unclear	Low	Unclear
Gopal et al. (52)	Unclear	Unclear	High	Unclear	High	Low	Unclear	Unclear	Low	Unclear	Low	Low
Greeson et al. (63)	Low	Unclear	High	Low	Low	Low	Low	Unclear	Low	High	Unclear	Unclear
Chang (53)	Low	Unclear	High	Unclear	High	Low	Unclear	Unclear	Low	Unclear	Unclear	Unclear
Nemati (54)	Unclear	Unclear	High	High	High	Low	Unclear	Unclear	Unclear	Unclear	Unclear	Unclear
Gopal et al. (52)	Low	Low	High	High	High	High	High	Low	Low	Unclear	Low	Unclear
Paholpak et al. (61)	Unclear	Unclear	High	High	Unclear	Low	Unclear	Unclear	High	Unclear	Low	High
Ratanasiripong et al. (66)	Unclear	Unclear	High	High	High	Unclear	Unclear	High	Low	Unclear	Low	Unclear
Shankarapillai et al. (55)	Unclear	Unclear	High	High	High	Low	Unclear	Low	Low	Low	High	Low
Shapiro et al. (32)	Unclear	Unclear	High	High	High	Low	Unclear	Low	Low	Unclear	Low	Unclear
Sharma et al. (56)	Unclear	Unclear	High	High	High	Unclear	Unclear	Unclear	Low	Unclear	Low	Low
Shearer et al, (67)	Unclear	Unclear	High	Unclear	High	Low	Unclear	Unclear	Low	Unclear	High	Unclear
van Dijk et al. (60)	Low	High	High	Low	High	Low	Low	Low	Low	Low	Unclear	Unclear
Yazdani et al. (68)	High	Unclear	High	High	High	Low	High	Unclear	Low	Unclear	Low	Low
Chang (50)	Unclear	Unclear	High	Unclear	Unclear	Low	Unclear	Unclear	High	Unclear	Unclear	Unclear
Chen et al. (29)	Low	Unclear	High	Unclear	High	Low	Unclear	Unclear	Low	Unclear	Low	High
Erogul et al. (58)	Low	Low	High	Unclear	High	Low	High	Unclear	Low	Unclear	Low	High
Kang et al. (65)	High	Unclear	High	High	Low	High	High	Low	High	Unclear	Low	High
Kvillemo et al. (64)	Low	High	High	High	Unclear	High	Low	Low	Low	High	High	Low
Malathi and Damodaran (48)	Unclear	Unclear	High	Unclear	Unclear	Unclear	Unclear	Unclear	Unclear	Unclear	Low	Unclear
Song and Lindquist (12)	Unclear	Unclear	High	High	Unclear	Low	Low	Unclear	Low	Unclear	Low	Unclear
Tloczynski (49)	Unclear	Unclear	High	Unclear	Unclear	High	High	Unclear	Unclear	Unclear	Low	Low

We conducted subgroup analyses on each risk of bias category but did not identify any significant association between the risk of bias and effect size. A subgroup analysis comparing “high” and “low” risk did not identify a significant difference *p* = 0.80. A further bivariate analysis on total risk of bias score and effect size did not identify significant subgroup differences either (coefficient: 0; 95% CI: −0.08 to 0.09; *p* = 0.91). Because almost all studies had a high risk of bias we were unable to assess the impact of risk of bias on the outcomes as we had too little power to identify differences between the groups.

### Publication Bias

**Figure 3:**
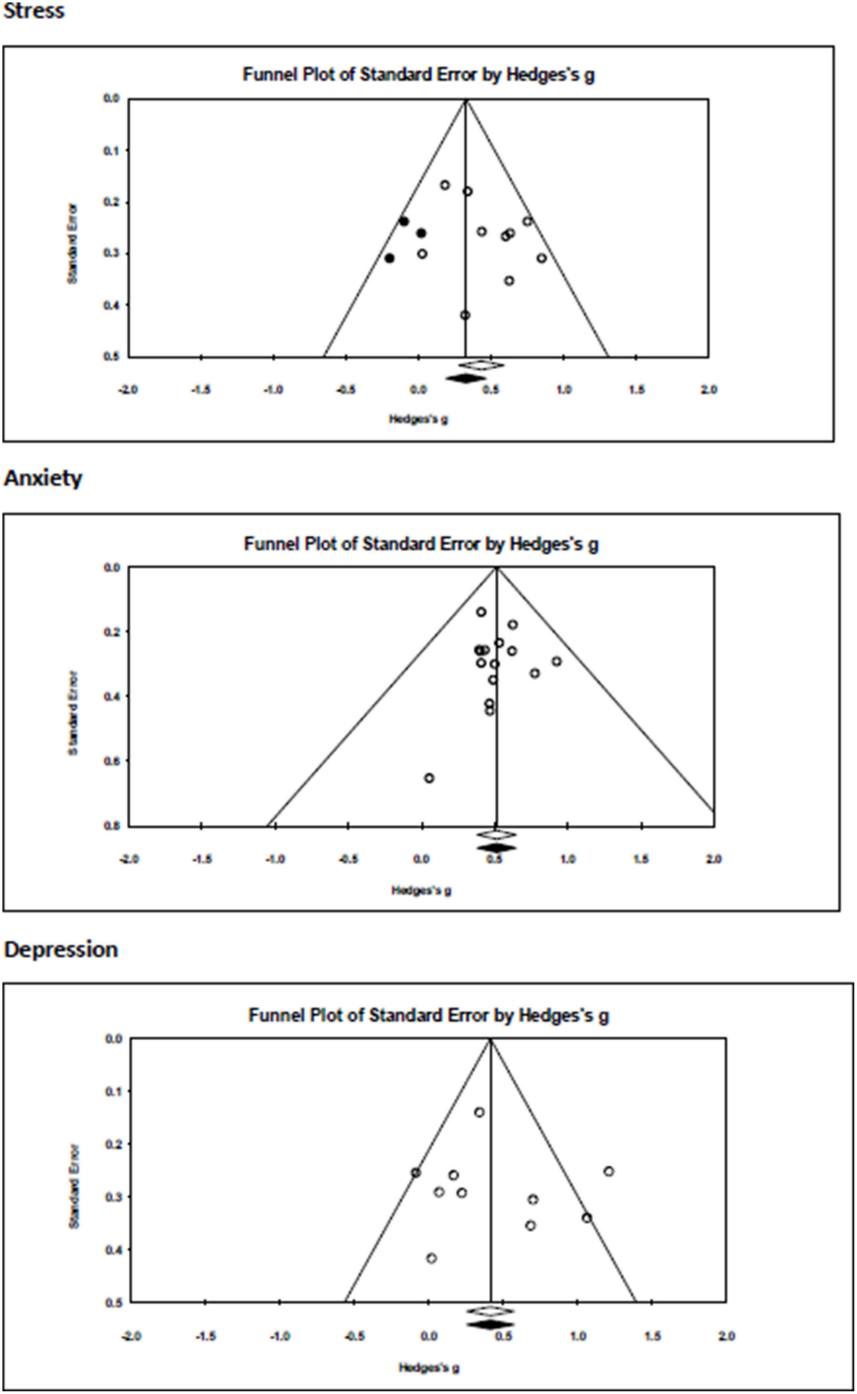
Visual inspection of the funnel plots for stress, anxiety, and depressive symptoms.

A visual inspection of the funnel plots for three separate outcomes (Figure 3) indicated no indication for publication bias for depression and anxiety but some risk of publication bias for the effects of mindfulness, yoga and meditation interventions on stress. Duval and Tweedie's trim and fill procedure under the random effects model did not impute any study effects, and the mean effect size remained unchanged except for studies assessing the effects of the intervention on stress from *g* = 0.44 (95%CI: 0.28–0.59) to *g* = 0.34 (95%CI: 0.16–0.51). The Egger's test did not find evidence for significant asymmetry of the funnel plot (*p* = 0.12; Intercept: 1.47, 95% CI: −1.25 to 4.18).

## Discussion

### Summary of Main Findings

In this study, we set out to assess the efficacy of mindfulness, meditation, and yoga on student mental health and academic achievement. This is the first study to assess the effects of meditation-based interventions in this population across all outcomes specified above.

We found moderate positive effects for mindfulness, yoga or meditation-based interventions on symptoms of depression, anxiety, and stress, however, the quality of the studies included in this review was low.

Subgroup analyses did not identify any differences were between yoga, mindfulness or meditation interventions. Two studies collected academic achievement data, making it impossible to render conclusions on the effects of such interventions on academic achievement. Similar to other reviews on mindfulness in this population, there were few studies with a long term follow up (23).

### Comparison to Previous Literature

When comparing our results to the previous literature on other interventions such as CBT or exercise, we find few studies that cover the same age range, inclusion criteria, similar outcomes and vs. similar controls.

From the few comparisons that we are able to make, we find that effects for CBT and exercise in non-clinical populations are more similar to our results compared to effects found in clinical interventions. A recent meta-analysis on preventative interventions for depression found g = 0.53 (95% CI: 0.38–0.68) for CBT (70). The effects of CBT on anxiety in a mixed (clinical and non-clinical population) are also within the range of our results *g* = 0.43 (95% CI: 0.14–0.73) (71). Effects of exercise on depression in non-clinical adolescent populations compared to control were not significant d = −0.52, (95% CI: −1.30 to 0.26) (72). For clinical populations, we identified slightly larger effect sizes for psychological treatment *g* = 0.89 (95% CI: 0.66–1.11) (73) and for exercise on depression g = −0.72, (95% CI: −1.15 to −0.30) (74).

In contrast, internet interventions for clinical and non-clinical populations overall had lower effects for depression *g* = 0.18, (95% CI: 0.08–0.27), anxiety *g* = 0.27 (95% CI: 0.13–0.40), and stress *g* = 0.20, (95% CI: 0.02–0.38) (75). Finally, comparing these results to mindfulness in students, mindfulness had somewhat similar effects depression in a previous analysis in a non-clinical sample *g* = 0.31 (95% CI: 0.15–0.42) (70). In another meta-analysis amongst healthy individuals, MBSR showed higher effect sizes across stress *g* = 0.74 (95% CI = 0.41–1.07), depression *g* = 0.80 (95% CI = 0.49–1.12), and anxiety *g* = 0.64 (95% CI: 0.33–0.94). However, across outcomes a subgroup analysis found more similar results for students *g* = 0.47 (95% CI = 0.30–0.64) (76).

To summarize, it seems our results are somewhat more in line with studies conducted in non-clinical samples compared to a clinical sample. Due to the heterogeneity found in the meta-analytic literature, it is not yet possible to compare exercise vs. mind-body interventions in a vis-à-vis manner although the above might give an indication of the comparative effectiveness of interventions.

Other similarities with previous include the high risk of bias which was similarly high in reviews on yoga (18, 77), exercise and psychotherapy (73, 74). A mixed risk of bias was found for internet interventions (75), it might be that such interventions carry a slightly lower risk to bias as blinding of participants and personnel might be easier compared to a face to face intervention.

Other similarities with previous include the high risk of bias which we noted, which was similar to reviews on yoga (18, 77), exercise and psychotherapy (73, 74). A mixed risk of bias was found for internet interventions (75), it might be that such interventions carry a slightly lower risk to bias as blinding of participants and personnel might be easier compared to a face to face intervention.

In addition, no adverse events were recorded and intervention elements were inadequately reported on, which is a common feature of the evidence base for yoga and meditation (27, 77–79).

### Comparison to Active Controls

In line with prior research on meditation programs, there was no evidence that meditation, yoga or mindfulness was more effective than active control (52, 76, 80). We had a limited number of subgroups so were unable to segment our analysis by active treatment (drugs, exercise or CBT) or active non-specific control (27). All our controls were deemed specific controls and thus are in line with (52) who found that meditation-based interventions were only more effective when compared to non-specific active control.

Research on psychotherapy in a similar population found that effects were also not more effective compared to active (specific) control (73). For exercise, these interventions were more effective compared to placebo control although further comparison with specific controls was not possible (74). Similar results were found for interventions (75) and other mental health interventions (81). The above summary indicates that the results are in line with previous research on both mindfulness and meditation interventions and behavioral interventions. As far as we are aware, we have not yet identified similar comparisons to specific or non-specific control conditions as detailed in Goyal et al. (27) for behavioral interventions, which would be an interesting element to explore in future research.

### Length and Duration

We did not find that the number of hours of meditation, yoga, and mindfulness is associated with effect size. Other meta-analyses on internet interventions did not find an effect on treatment length (weeks) (75). Meta-analyses with a majority of interventions based on CBT did find that interventions with a longer duration (hours) were associated with effect size (71, 81). In our analysis, we included both home practice as well as treatment duration, whilst the above analyses only specified the amount of treatment, this difference in coding might have altered our results. Also we were not always able to exactly specify the amount of home practice, which might again affect the validity of our result.

### Limitations

In contrast to psychotherapeutic interventions, there remains a substantial degree of uncertainty about the robustness of our effect. Our study found that most studies were of lower quality and those improvements to reporting and procedures of studies in this area are required. High risk of bias is a concern as this impacts the validity of the findings and our confidence in making any conclusions from our analysis (82). Whilst risk of bias was not associated with effect size, this might be due to low power to detect such a difference. Due to the high risk of bias in most studies, it is difficult to determine the true value of these approaches and more rigorous research is clearly needed to assess for improving mental health in tertiary education. Whilst the quality of studies across behavioral interventions is low, we note that there are specific elements to meditation, yoga and mindfulness interventions that warrant improvement.

We found that intervention effects diminished when compared to active control, which implies that non-specific intervention elements such as peer-support, or activity scheduling, might have driven our results. To further study the differential effect of mindfulness, meditation, and yoga, a comprehensive typology of intervention elements is necessary. A shared understanding of differential intervention elements will allow development of adequate placebo control conditions to identify whether the contribution of mindfulness, meditation or yoga improves mental health or whether they are equally effective as non-specific placebo interventions.

Interestingly, we did not find an association between the exposure to yoga, mindfulness or meditation through treatment or home practice and effect size. This is surprising given the premise that yoga and mindfulness are seen as practices that improve over time (83). One might then expect to see a positive association between the amount of recent mindfulness practice and effect sizes. There is a caveat however as the amount of home practice was not always reported on consistently, thus due to a lack of clear reporting, we may have been unable to accurately estimate the exposure to such interventions.

### Conclusions

To improve the evidence base, the conduct and reporting of studies on meditation, yoga, and mindfulness needs to be more rigorous to allow the delivery of results that are closer to their empirical truth. Furthermore, we recommend that a common typology for meditation, yoga and mindfulness interventions is developed and that future research includes comparisons between active placebo and control. This will allow us to determine the true differential effects between mindfulness, meditation, and yoga and in comparison, to other approaches to improve mental health. Ultimately, this will allow us to further our understanding of delivering effective non-clinical solutions for protecting and promoting mental health in student populations.

## Author Contributions

JB selected and extracted the studies, conducted the analysis, prepared tables and prepared the first draft for review. YA selected and extracted the studies and prepared the tables for the first draft. Revising work critically for important content and accuracy. MH led on data acquisition and design of the work. Contributed strongly to the methods section and revised work critically after the first draft was prepared. EK led on conception and design of the work. Critically revised the first and second draft for accuracy and intellectual content. SG critically revised for intellectual content and reviewed first and second draft. Contributed to analysis plan. CB critically revised for intellectual content and contributed to plan of analysis. PC led on conception and design and provided support with analytical decisions in analysis stage critically reviewed the first and second draft for intellectual and conceptual relevance. DE led on conception and design of the work, critically revised the first and second draft for intellectual and conceptual relevance.

### Conflict of Interest Statement

JB is employed by the Mental Health Foundation which offers an online Mindfulness Based Cognitive Therapy course titled BeMindful. She is not directly affiliated to the delivery or development of this programme. The remaining authors declare that the research was conducted in the absence of any commercial or financial relationships that could be construed as a potential conflict of interest.
