# Supplementary material for: The Effects of Meditation, Yoga, and Mindfulness on Depression, Anxiety, and Stress in Tertiary Education Students: A Meta-Analysis
Source: Front Psychiatry. 2019 Apr 24;10:193. doi: 10.3389/fpsyt.2019.00193 (PMC6491852; doi:10.3389/fpsyt.2019.00193)
Supplement: Supplementary file 1 [file Data_Sheet_1.PDF]

Search		Query
#77	Add	Search (#19 AND #33 AND #76)
#76	Add	Search (#1 OR #2 OR #3 OR #4 OR #5 OR #6 OR #7 OR #8 OR #9 OR #10 OR #11 OR #12 OR #13 OR #34 OR #35 OR #36 OR #37 OR #38 OR #39 OR #40 OR #41 OR #42 OR #43 OR #44 OR #45 OR #46 OR #47 OR #48 OR #49 OR #50 OR #51 OR #52 OR #53 OR #54 OR #55 OR #56 OR #57 OR #58 OR #59 OR #60 OR #61 OR #62 OR #63 OR #64 OR #65 OR #66 OR #67 OR #68 OR #69 OR #70 OR #71 OR #72 OR #73 OR #74 OR #75)
#75	Add	Search (anxiety OR fear OR dental anxiety OR panic[MeSH Terms])
#74	Add	Search (brain[Text Word] AND doping[Text Word])
#73	Add	Search pornography*[Text Word]
#72	Add	Search (problematic*[Text Word] AND internet*[Text Word])
#71	Add	Search (internet*[Text Word] AND addiction[Text Word])
#70	Add	Search (social[Text Word] AND media[Text Word])
#69	Add	Search (cognitive AND enhance*)
#68	Add	Search neuroenhance*[Text Word]
#67	Add	Search nicotine*[Text Word]
#66	Add	Search (test*[Text Word] AND anxiety[Text Word])
#65	Add	Search self-efficacy[Text Word]
#64	Add	Search (time[Text Word] AND management[Text Word])
#63	Add	Search time manag*[Text Word]
#62	Add	Search (social*[Text Word] AND skill*[Text Word])
#61	Add	Search (social[Text Word] AND competence[Text Word])
#60	Add	Search stress*[Text Word]
#59	Add	Search resilience[Text Word]
#58	Add	Search (health[Text Word] AND behaviour[Text Word])
#57	Add	Search (health[Text Word] AND behavior)
#56	Add	Search (behaviour[Text Word] AND modification[Text Word])
#55	Add	Search (behavior[Text Word] AND modification[Text Word])
#54	Add	Search (behaviour[Text Word] AND change[Text Word])
#53	Add	Search (behavior[Text Word] AND change[Text Word])
#52	Add	Search eating*[Text Word]
#51	Add	Search weight*[Text Word]
#50	Add	Search procrastination*[Text Word]
#49	Add	Search rumination*[Text Word]
#48	Add	Search fear*[Text Word]
#47	Add	Search worry*[Text Word]
#46	Add	Search obesity*[Text Word]
#45	Add	Search exercise*[Text Word]
#44	Add	Search (physical[Text Word] AND activity[Text Word])
#43	Add	Search smoking*[Text Word]
#42	Add	Search marijuana[Text Word]
#41	Add	Search cannabis[Text Word]
#40	Add	Search substance*[Text Word]
#39	Add	Search drug*[Text Word]
#38	Add	Search alcohol*[Text Word]
#37	Add	Search HIV[Text Word]
#36	Add	Search condom*[Text Word]
#35	Add	Search (sexual*[Text Word] AND health[Text Word])

#34	Add	Search **(mental disorders OR anxiety disorders OR bipolar and related disorders OR disruptive, impulse control, and conduct disorders OR dissociative disorders OR elimination disorders OR feeding and eating disorders OR mood disorders OR motor disorders OR neurocognitive disorders OR neurodevelopmental disorders OR neurotic disorders OR paraphilic disorders OR personality disorders OR schizophrenia spectrum and other psychotic disorders OR sexual dysfuntions, psychological OR sleep wake disorders OR somatoform disorders OR substance-related disorders OR trauma and stressor related disorders[MeSH Terms])**
#33	Add	Search **(#32 NOT #31)**
#32	Add	Search **(#20 OR #21 OR #22 OR #23 OR #24 OR #25 OR #28 OR #29 OR #30)**
#31	Add	Search **(animals [mh] NOT (animals [mh] AND humans [mh]))**
#30	Add	Search **clinical trials as topic [mh]**
#29	Add	Search **trial[Title]**
#28	Add	Search **(#26 AND #27)**
#27	Add	Search **(waitlist*[Title/Abstract] OR wait* list*[Title/Abstract] OR treatment as usual[Title/Abstract] OR TAU[Title/Abstract])**
#26	Add	Search **(control*[Title/Abstract] OR group* 1[Title/Abstract])**
#25	Add	Search **placebo*[Title/Abstract]**
#24	Add	Search **randomly[Title/Abstract]**
#23	Add	Search **randomised[Title/Abstract]**
#22	Add	Search **randomized[Title/Abstract]**
#21	Add	Search **controlled clinical trial[Publication Type]**
#20	Add	Search **randomized controlled trial[Publication Type]**
#19	Add	Search **(#14 OR #15 OR #16 OR #17 OR #18)**
#18	Add	Search **(tertiary*[Title/Abstract] AND education*[Title/Abstract])**
#17	Add	Search **(college*[Title/Abstract] AND student*[Title/Abstract])**
#16	Add	Search **(undergraduate*[Title/Abstract] AND student'[Title/Abstract])**
#15	Add	Search **(university*[Title/Abstract] AND student*[Title/Abstract])**
#14	Add	Search **student*[Text Word]**
#13	Add	Search **internet*[Text Word]**
#12	Add	Search **online*[Text Word]**
#11	Add	Search **web*[Text Word]**
#10	Add	Search **online therap*[Text Word]**
#9	Add	Search **therap*[Text Word]**
#8	Add	Search **psychotherap*[Text Word]**
#7	Add	Search **program*[Text Word]**
#6	Add	Search **psychoeducation*[Text Word]**
#5	Add	Search **course*[Text Word]**
#4	Add	Search **counsel*[Text Word]**
#3	Add	Search **treatment*[Text Word]**
#2	Add	Search **training*[Text Word]**
#1	Add	Search **intervention*[Text Word]**
