# Supplementary material for: The Effects of Meditation, Yoga, and Mindfulness on Depression, Anxiety, and Stress in Tertiary Education Students: A Meta-Analysis
Source: Front Psychiatry. 2019 Apr 24;10:193. doi: 10.3389/fpsyt.2019.00193 (PMC6491852; doi:10.3389/fpsyt.2019.00193)
Supplement: Supplementary file 2 [file Data_Sheet_2.docx]

**Appendix 2. Alternative medicine treatments in tertiary education students. Overview of study subgroups generated after title and abstract screening**

| **Screening criterion** | *“Practices that are not part of standard care”*  *“A broad set of health care practices that* […] *are not integrated into the dominant health care system”*^[[1]](#footnote-1)^ (WHO, 2000; [Link](https://tinyurl.com/y96du9lr)) | |
| --- | --- | --- |
| **Studies included after title, abstract screening** | | *k* = 181 |

| **Subgroup** | **Commentary** | *k* |
| --- | --- | --- |
|  |  |  |
| Aromatherapy/Herbotherapy | e.g. lavender, rose, sage oil | 27 |
| Meditation | - | 22 |
| Yoga | - | 18 |
| Acupuncture/Acupressure/Moxibustion | - | 18 |
| Mindfulness | - | 18 |
| Art/Dance Therapy | e.g. Painting, Mandala Drawing, African Dance | 13 |
| Tai-Chi / Qi-Gong / Baduanjin | Combination of body movement, breathing exercises | 9 |
| Hypnosis | Including Self-Hypnosis | 8 |
| Religious behavior | e.g., praying, “counting blessings” | 7 |
| Vitamin Preparations | e.g. Vitamin C, Vitamin B, Vitamin B1; studies with psychological outcomes | 5 |
| Massage | - | 3 |
| Humor | - | 3 |
| Other Alternative Medicine | e.g., “Emotional Freedom Techniques” Therapy, Vestibular Stimulation, “Quantum Healing”, Forest therapy | 30 |
|  |  |  |

1. The US National Science Board (2002) refers to Alternative Medicine as “all treatments that have not been proven as effective using scientific methods” ([Link](http://tinyurl.com/ycu2nq2w)). This definition makes an a priori assumption on the efficacy of alternative treatments which has yet to be proven. Thus, criteria above were applied to screen for overall fit. [↑](#footnote-ref-1)
